# Relationship between Expression of Plasma lncRNA-HEIH and Prognosis in Patients with Coronary Artery Disease

**DOI:** 10.1155/2021/5662080

**Published:** 2021-12-31

**Authors:** Zhenying Zhang, Sushuang Nan, Xiujuan Duan, Lizhong Wang, Xiaojing Sun, Haiying Zheng

**Affiliations:** ^1^Cardiac Rehabilitation Center, Beijing Rehabilitation Hospital, Capital Medical University, Beijing, China; ^2^Department of Cardiology, The Eighth People's Hospital of Hengshui City, Hengshui, China; ^3^Department of Cardiovascular, Affiliated Hospital of Inner Mongolia Medical University, Hohhot, China

## Abstract

**Objective:**

We aimed to investigate the expression of long noncoding RNA- (lncRNA-) HEIH in patients with coronary artery disease (CAD) and its impact on patients' prognosis. *Patients and Methods*. From July 2015 to December 2018, 250 patients who underwent coronary angiography, including 50 in the control group and 150 in the CAD group, were collected for detection of the expression of lncRNA-HEIH by real-time quantitative polymerase chain reaction (qPCR). The severity of CAD was evaluated through SYNTAX scoring system. In addition, these patients with CAD were followed up for 3 years, and the major cardiac adverse events such as myocardial infarction and revascularization were recorded.

**Results:**

The expression of lncRNA-HEIH in plasma of patients with CAD was remarkably higher than that in the control subjects and was verified to be relevant to the severity of CAD. Meanwhile, it was found that CAD patients with high expression of lncRNA-HEIH had higher rates of dyslipidemia as well as CAD family history and higher overall incidence of major cardiac adverse events than those with low expression of lncRNA-HEIH.

**Conclusions:**

lncRNA-HEIH expression is upregulated in the plasma of CAD patients, which is capable of affecting the prognosis of patients.

## 1. Introduction

Coronary artery disease (CAD) is one of the main causes of death in the world at present [1]. About 5% of Asians over the age of 18 suffer from CAD; what is more, its prevalence is still increasing [2]. Despite the rapid development of cardiovascular interventional techniques, a significant proportion of patients with coronary heart disease have not been accurately diagnosed at an early stage and cannot be effectively treated during the onset of myocardial infarction, causing severe damage to myocardial cells and ultimately leading to myocardial cell necrosis, apoptosis, hypertrophy, or fibrosis [3, 4].

Long noncoding RNA (lncRNA) refers to a nonproteinized nucleotide with a length greater than 200, which can regulate the expression level of genes in the form of RNA at various levels, so it has a lot of complex secondary structures [5]. It can be involved in a variety of important regulatory processes such as intracellular transport, transcriptional activation and interference, and chromosome silencing, through multiple mechanisms such as cell differentiation regulation, gene imprinting, splicing regulation, degradation, chromatin remodeling, and mRNA translation regulation [6, 7].

It has been confirmed that lncRNA plays a pivotal role in a number of biological processes, including fat metabolism, atherosclerosis formation, embryonic development, and tumor occurrence [8]. Recent studies have demonstrated that lncRNAs have high tissue specificity in myocardial tissue. Moreover, a genome-wide analysis of cardiac transcription following myocardial infarction revealed that a large number of cardiac-specific lncRNAs have unique regulatory and functional properties for pathophysiological processes such as myocardial remodeling and myocardial regeneration. This finding means that cardiac-specific lncRNA may serve as a targeting molecule and biomarker for heart development and disease [9]. lncRNA-HEIH is a newly discovered long-chain noncoding RNA with high expression level in liver cancer tissues, which was confirmed to be correlated with high recurrence rate of liver cancer patients and thus can be used as one of the predictors of survival after treatment [10]. Even many reports demonstrated that lncRNA-HEIH plays an important role in the prognosis of patients in different kinds of cancers. However, there is no evidence showing the association between lncRNA-HEIH and nontumor diseases. We attempted to detect the expression level of lncRNA-HEIH in the serum of CAD patients and tried to explore the potential association with the prognosis. Therefore, in this study, we detected serum lncRNA-HEIH level of CAD patients and explored its relationship with the occurrence of major clinical adverse events, which may provide a basis for risk prediction of CAD events.

## 2. Patients and Methods

### 2.1. Research Object

Patients who underwent coronary angiography in Beijing Rehabilitation Hospital, Capital Medical University, were randomly selected from July 2015 to December 2018 including 50 patients in the control group (negative control (NC)) and 150 patients in the CAD group. Diagnostic criteria are as follows. Patients in the control group were admitted to the hospital with precardiac discomfort, and no obvious stenosis in the coronary arteries was found by coronary angiography. For patients with CAD, results of coronary angiography were analyzed by computerized quantitative software, and the degree of stenosis was calculated using the diameter method. According to the American College of Cardiology coronary vascular scoring criteria, one or more branches, including the left anterior descending artery, the left coronary artery trunk, the circumflex artery, and the right coronary artery, occurred ≥he circumflex can be diagnosed as CAD. This study was approved by the Ethics Committee of the hospital.

### 2.2. Plasma Collection

Fasting blood samples of each subject were collected before coronary angiography and anticoagulated with ethylenediaminetetraacetic acid (EDTA) dipotassium salt in the morning. After centrifugation at 3000 r/min at 4°C for 15 min, plasma was collected and stored at -80°C until assayed.

### 2.3. RNA Extraction

Total RNA in serum was extracted by TRIzol method (Invitrogen, Carlsbad, CA, USA) and then reverse transcribed into complementary deoxyribonucleic acid (cDNA). RT-PCR was performed according to the TaqMan RNA Reverse Transcript Kit protocol. The reaction system volume was in total 25 *μ*l, predenaturation at 95° for 5 min, denaturation at 95° for 30 sec, annealing at 60° for 45 sec, extension at 72° for 3 min, with 35 cycles, and then extension at 72° for 5 min. PCR products were stored at 4°. Quantitative analysis was carried out using the ABI 7500 fluorescence PCR amplification instrument (Applied Biosystems; Thermo Fisher Scientific, Inc.). lncRNA-HEIH primer sequence: forward: 5′-CCTCTTGTGCCCCTTTCT-3′, reverse: 5′-AGGTCTCATGGCTTCTCG-3′; internal reference glyceraldehyde 3-phosphate dehydrogenase (GAPDH) upstream primer forward: 5′-TGTTGCCATCAATGACCCCTT-3′, downstream primer reverse: 5′-CTCCACGACGTACTCAGCG-3′.

### 2.4. SYNTAX Score

The angiographic results of each patient were scored using the SYNTAX webpage (http://www.syntaxscore.com/), and CAD patients were subdivided into three groups.

### 2.5. Follow-Up Information

After discharge, major adverse cardiac events (nonfatal myocardial infarction, death, revascularization, etc.) in all patients were recorded by telephone or outpatient follow-up at least once a month. Determination of myocardial infarction: chest pain lasting more than 20 minutes or new abnormal changes in electrocardiogram accompanied by abnormal increase of troponin. Determination of revascularization: retreatment of the blood vessel after surgery or intervention or the treatment of new stenosis of the blood vessel. The follow-up period lasted for 3 years.

### 2.6. Statistical Analysis

Analysis was performed using Statistical Product and Service Solutions (SPSS) 20.0 statistical software (IBM, Armonk, NY, USA). The measurement data were expressed as mean ± standard deviation, the independent sample *t*-test was used for comparison between the two groups, and the variance analysis was used for comparison of multiple groups. The count data was compared by the *χ*^2^ test; ^∗^*p* < 0.05.

## 3. Results

### 3.1. High Expression of lncRNA-HEIH in Plasma of CAD Patients

**Figure 1:**
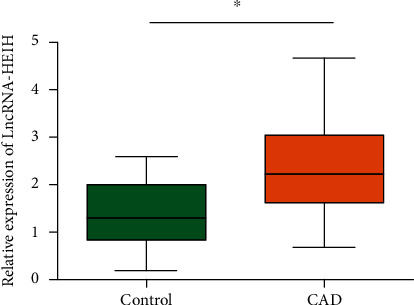
High expression of lncRNA-HEIH in plasma of CAD patients. The expression of lncRNA-HEIH in plasma of patients with CAD was remarkably higher than that in plasma of control subjects.

To determine the role of lncRNA-HEIH in CAD, we examined its expression in plasma by qRT-PCR, and lncRNA-HEIH was found to be remarkably increased in plasma of CAD patients compared with that in the control group (Figure 1), indicating that lncRNA-HEIH may affect the development of CAD.

### 3.2. Comparison of Plasma lncRNA-HEIH Levels in CAD Patients

Based on the average expression level of lncRNA-HEIH in all CAD patients, we divided CAD patients into the lncRNA-HEIH high-expression group (*n* = 103) and the low-expression group (*n* = 47). The *χ*^2^ test of the clinical parameters of CAD patients revealed that the rate of CAD family history and dyslipidemia was closely relevant to lncRNA-HEIH expression in the serum of CAD patients, but no significant relevance was found between its level and some other indicators, such as gender, age, BMI, smoking, and hypertension history (Table 1). The above observations confirmed that lncRNA-HEIH expression is associated with a family history of CAD and dyslipidemia incidence.

### 3.3. Correlation between Plasma lncRNA-HEIH Levels and Severity of CAD

**Figure 2:**
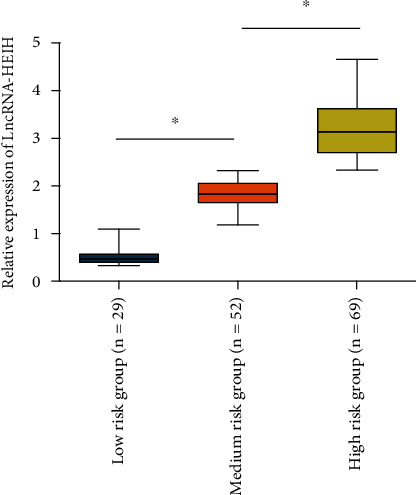
Analysis of correlation between plasma lncRNA-HEIH levels with severity of CAD. CAD patients were divided into the low-risk group (SYNTAX score < 23; *n* = 29), the intermediate-risk group (SYNTAX score 23-32; *n* = 52), and the high-risk group (SYNTAX score > 32; *n* = 96) according to SYNTAX score. The level of lncRNA-HEIH in plasma was detected by qRT-PCR. The lncRNA-HEIH in the middle-risk group was remarkably higher than that in the low-risk group. The lncRNA-HEIH in the high-risk group was also remarkably higher than that in the middle-risk group.

To further clarify the impact of lncRNA-HEIH on CAD, according to the SYNTAX score, we divided CAD patients into the low-risk group (SYNTAX score < 23; *n* = 29), the intermediate-risk group (SYNTAX score 23-32; *n* = 52), and the high-risk group (SYNTAX score > 32; *n* = 96) and then verified through qPCR assay that patients in the high-risk group contained the highest expression of lncRNA-HEIH (Figure 2), further demonstrating that lncRNA-HEIH is able to affect the severity of CAD patients.

### 3.4. Effect of lncRNA-HEIH on the Occurrence of Major Clinical Adverse Events in CAD Patients

To investigate the impact of lncRNA-HEIH on the prognosis of patients with CAD, we performed 3 years of follow-up and found that patients with high expression of lncRNA-HEIH had a higher overall incidence of major adverse cardiac events and a higher death rate (*p* < 0.05, Table 2), suggesting that highly expressed lncRNA-HEIH is not conducive to the prognosis of CAD patients.

## 4. Discussion

Coronary artery disease is one of the major diseases threatening health. The occurrence of CAD can be induced by the deposition of cholesterol and fat in the artery, which narrows the channel of blood into the heart and leads to thrombosis. Insufficient coronary artery blood supply can easily lead to angina pectoris and myocardial infarction [11]. Although percutaneous coronary intervention (PCI) and coronary artery bypass grafting (CABG) have been confirmed to be effective heart artery reconstruction methods for clinical CAD treatment, some studies [12–14] have suggested that the limited effect of CABG and PCI on improving the survival and the high cost limit their use in clinical practice. Since early clinical intervention on CAD patients can remarkably prolong the survival of patients, finding a less invasive examination method with higher accuracy has become the focus of clinical attention [15].

Research findings in the last decade indicate that, driven by the rapid emergence of genomics and proteomics, circular lncRNAs have become the most attractive and promising biomarkers in cardiovascular diseases. In a study published by Li et al. [16] on microarray of mouse model, they analyzed the expression level of lncRNAs in different body fluids (mainly whole blood and plasma) and found a more abundant gene expression profile compared with specific heart tissue lncRNAs. For example, ANRIL [9] and CDKN2A/B [17] are closely associated with atherosclerosis (especially coronary arteries), while MIAT [18] and LIPCAR [19] play an irreplaceable role in myocardial infarction and heart failure.

lncRNA-HEIH is a long-chain noncoding RNA specifically expressed in liver cancer tissues, which has certain reference value for recurrence and prognosis of liver cancer [20]. The main novelty of this research is that we investigated for the first time the expression of l lncRNA-HEIH in patients with coronary artery disease (CAD) and its impact on patients' prognosis. In our research, lncRNA-HEIH levels were found to be markedly elevated in CAD patients compared to control subjects, and this increase was positively correlated with the severity of CAD. In addition, statistical analysis revealed that patients with a family history of CAD and dyslipidemia had higher expression levels of lncRNA-HEIH. The 3-year follow-up results also suggested that highly expressed lncRNA-HEIH induced a higher incidence of major adverse cardiac events, which indicates a poor prognosis. This study may provide further evidence for CAD diagnosis and prognosis assessment. There are still some limitations in this present study. The sample was not big and the follow-up was not long enough to conduct the further analysis. Also, we did not perform the molecular cellular experiments to explore the potential mechanism. In our future study, we plan to conduct the in vitro and in vivo assays for the further verification.

## 5. Conclusions

lncRNA-HEIH is highly expressed in serum of CAD patients, which is not conducive to the prognosis of patients.

## Figures and Tables

**Table 1:** Plasma lncRNA-HEIH levels in CAD patients.

Variable	*n*	High level (*n* = 103)	Low level (*n* = 47)	*χ* ^2^	*p*
Sex					
Male	27	22	5	2.513	0.168
Female	123	81	42		
Age					
<60	56	37	19	0.280	0.716
≥60	94	66	28		
BMI (kg/m^2^)					
<24	61	42	19	0.002	1.000
≥24	89	61	28		
Smoking					
No	72	45	27	2.447	0.158
Yes	78	58	20		
Family history of hypertension					
No	38	28	10	0.596	0.545
Yes	112	75	37		
Family history of CAD					
No	32	29	3	9.116	0.002^∗^
Yes	118	74	44		
Dyslipidemia					
No	58	34	24	4.436	0.047^∗^
Yes	92	69	23		

BMI: body mass index; ^∗^*p* < 0.05.

**Table 2:** Effect of lncRNA-HEIH on major clinical adverse events of CAD patients [*n* (%)].

Variable	High level (*n* = 103)	Low level (*n* = 47)	*χ* ^2^	*p*
Cardiac adverse event (*n* = 42)	37 (35.92%)	5 (10.64%)	10.23	0.001^∗^
Death (*n* = 15)	14 (13.59%)	1 (2.13%)	4.713	0.038^∗^
Nonfatal myocardial infarction (*n* = 17)	11 (10.68%)	6 (12.77%)	0.14	0.783
Vascular reconstruction (*n* = 23)	18 (17.48%)	5 (10.64%)	1.162	0.336
Restenosis (*n* = 19)	11 (10.68%)	8 (17.02%)	1.173	0.297
First stenosis (*n* = 6)	4 (3.88%)	2 (4.26%)	0.012	1.000

^∗^ *p* < 0.05.

## Data Availability

The datasets used and analyzed during the current study are available from the corresponding author on reasonable request.
